# Tracking spread of carbapenemase-producing Enterobacterales between humans and companion animals: successes and challenges

**DOI:** 10.3389/fcimb.2025.1730592

**Published:** 2026-01-20

**Authors:** Jaclyn M. Dietrich, Paula M. Snippes Vagnone, Jennifer L. Dale, Amanda L. Beaudoin, Abbey Ruhland, Leslie Kollmann, Stephen D. Cole

**Affiliations:** 1 Clinical Infectious Disease Laboratory, Department of Pathobiology, School of Veterinary Medicine, University of Pennsylvania, Philadelphia, PA, United States; 2 Minnesota Department of Health, St Paul, MN, United States; 3 College of Veterinary Medicine, University of Minnesota, St Paul, MN, United States

**Keywords:** carbapenemase-producing Enterobacterales, companion animals, one health, public health, veterinary medicine

## Abstract

Carbapenemase-producing Enterobacterales (CPE) pose a critical public health threat. Recent reports highlight that CPE emergence in companion animals mirrors that found in humans, underscoring the need for a One Health approach to investigating transmission routes. This Perspective article outlines an interdisciplinary model developed as part of the Centers for Disease Control and Prevention-funded Pathogen Genomics Center of Excellence at the Minnesota Department of Health (MDH) to investigate CPE transmission across human and animal populations. This represents one of the first operational One Health models linking companion animal and human CPE via whole genome sequencing (WGS) at a state-level public health laboratory. Using WGS, 94 companion animal isolates were characterized and revealed diverse genetic lineages from December 2022 through December 2024 in the USA. Genetically linked clusters were identified, including CPE isolates from companion animals and humans. One notable cluster linked human infections with CPE detected in a veterinary hospital. Despite the success of this approach to detect clusters, there were significant challenges, including investigation delays related to sequencing and epidemiology priorities and capacity, resource constraints, and human participant hesitancy. Our findings demonstrate the importance of integrating genomic data with clinical and epidemiological insights, fostering communication between veterinary and public health sectors, and expanding veterinary WGS infrastructure. Ultimately, we advocate for broader public health engagement in veterinary settings to mitigate antimicrobial resistance and improve surveillance of zoonotic transmission pathways.

## 1 Introduction

Carbapenemase-producing Enterobacterales (CPE), including *Klebsiella pneumoniae*, *Escherichia coli* and *Enterobacter cloacae* complex, are among the most urgent antimicrobial resistance (AR) threats according to the World Health Organization and Centers for Disease Control and Prevention (CDC) (World Health Organization, 2024; Centers for Disease Control and Prevention (CDC), 2022). Traditionally viewed as agents of hospital-associated infections (HAI) in humans, CPE have been reported to cause outbreaks in companion animals (DeStefano et al., 2025a, Cole et al., 2020, Silva et al., 2022, Nigg et al., 2019).

Within the last decade, whole genome sequencing (WGS), paired with bioinformatic techniques and workflows, has greatly expanded the ability to track the clonal spread of pathogens (Sherry et al., 2025). For example, cases of human campylobacteriosis were connected by WGS to *Campylobacter* transmission among puppies sold at pet stores throughout the USA (Francois Watkins et al., 2021). Open databases for sharing sequencing data or assemblies, such as the National Center for Biotechnology Information (NCBI), have also increased the capacity to connect bacterial isolates across human, animal, and environmental sources (National Center for Biotechnology Information (NCBI), 2025). These advances offer specific opportunities to track pathogens within a One Health context, which recognizes that the health of humans and animals is intertwined Ballash (Ballash et al., 2024).

In this Perspective article, we describe our interdisciplinary team’s successes and challenges in investigating the epidemiology of CPE across human and animal populations in the United States between December 2022 and December 2024. Here we aim to (1) describe the model of our One Health approach in investigating related CPE isolates across human and animal populations and (2) use a data subset to illustrate our experiences utilizing the described One Health model. Lastly, we (3) discuss opportunities that we see for unraveling the complexities of transmission and risk by integrating genomic and clinical epidemiology data.

## 2 One health model

**Figure 1:**
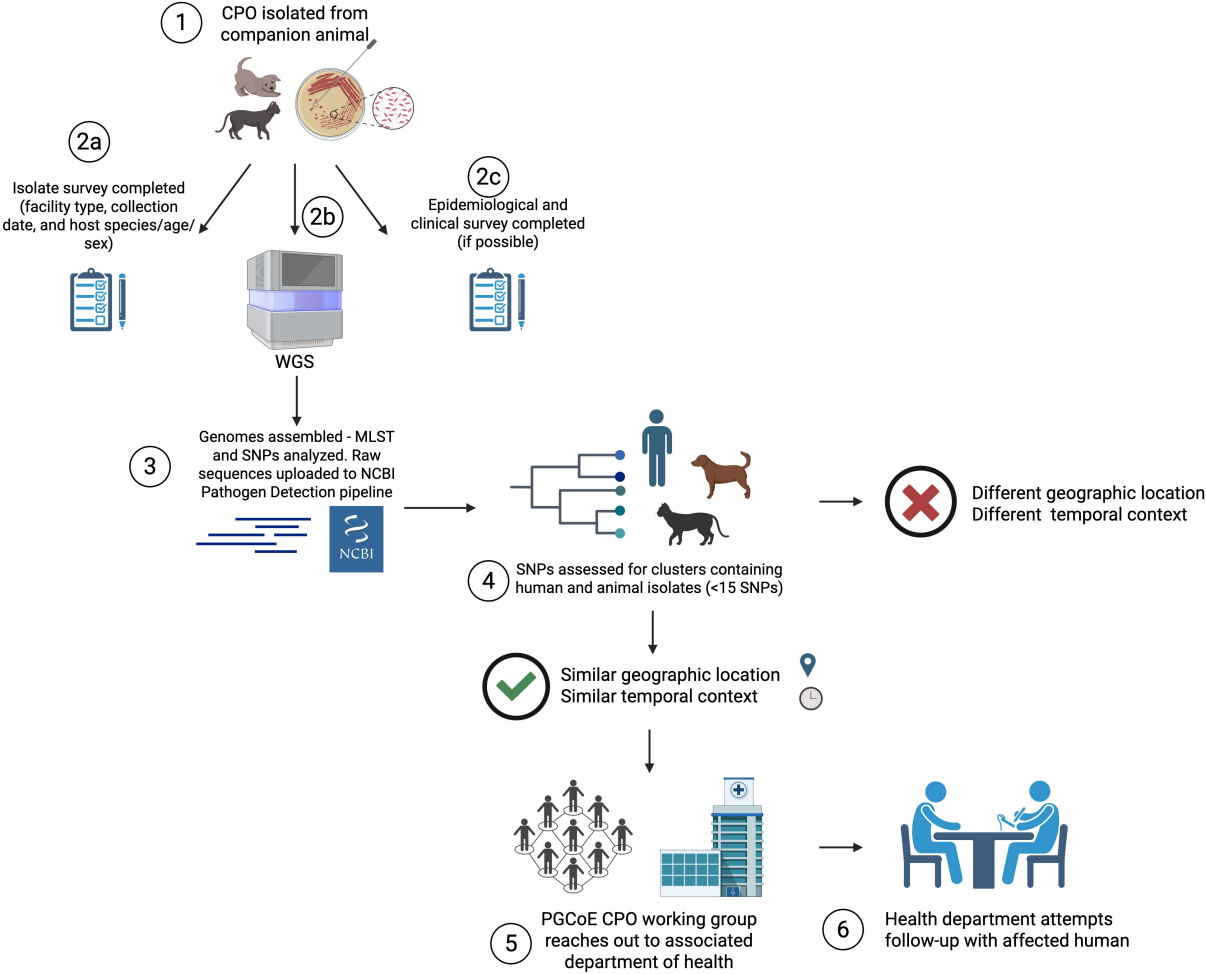
Outline of human-animal Carbapenemase-producing Organism (CPO) investigation model. Whole genome sequencing (WGS); multilocus sequence typing (MLST); single nucleoptide polymorphism (SNP); Pathogen Genomics Center of Excellence (PGCoE). Created in BioRender. Dietrich, J. (2025) https://BioRender.com/7tulsmj.

In 2022, we piloted an approach through the CDC Pathogen Genomics Centers of Excellence Network (PGCoE) to examine the relatedness of carbapenemase-producing organisms (CPO) isolated from animals and humans (**Figure 1**). The PennVet Clinical Infectious Disease Lab (CID) serves as a companion animal diagnostic laboratory for both the Ryan Veterinary Hospital at the University of Pennsylvania as well as a general microbiology reference diagnostic laboratory for veterinarians across the United States. Furthermore, the laboratory serves as a CPO reference laboratory through the Carbapenem Resistant Enterobacterales Animal Testing and Epidemiology (CREATE) Project for veterinarians and laboratories that may not have the bandwidth to perform confirmatory testing. Through these avenues, the PennVet CID acquired residual CPO isolates from companion animals and performed testing to confirm the presence or absence of carbapenemase(s) by the modified carbapenemase inactivation method (Pierce et al., 2017). Use of residual specimens and bacterial isolates is not considered animal research and is exempt from IACUC review at the University of Pennsylvania. A subset of organisms in the collection were sequenced immediately following isolation, and some archived isolates were sequenced retrospectively from an existing collection at the PennVet CID Lab. All CPO isolates retained by PennVet CID Lab between December 2022 and December 2024 were included in these investigations described hereafter. While the initial goals of this study were inclusive of all CPOs, currently all isolates have been identified as members of the Enterobacterales order and are specifically referred to as CPE within this article.

Once carbapenemase production was confirmed, the isolate was sent to the MDH Public Health Laboratory (MDH PHL), which serves as a CDC Antimicrobial Resistance (AR) Laboratory Network regional laboratory, and prepared for WGS. MDH PHL collected information related to submitted isolates into a REDCap project containing multiple survey instruments (Research Electronic Data Capture, Harris et al., 2009). Upon isolate submission, PennVet CID Lab entered data about isolate collection location (tertiary care facility, general practice, referral practice, animal rescue/shelter, or other), animal signalment (species, breed, age, and sex), and isolate details (date of collection, organism identification, clinical or screening specimen, specimen type, carbapenemase tests conducted and their results). When possible, epidemiological and clinical information about the source animal was entered by the institution that originally submitted the isolate to PennVet CID Lab into REDCap survey instruments (**Supplementary Informations 1**, **2**).

WGS was performed using Illumina MiSeq™ v2 or v3 (2x250 PE) chemistry followed by genome assembly, carbapenemase detection, and multilocus sequence type (MLST) determination using Spriggan 1.1.2. Single nucleotide polymorphism (SNP) analysis was performed on isolates with the same MLST using CFSAN as part of the Dryad 3.0.0 workflow, with a phylogenetic tree generated using RAxML (DeStefano et al., 2025b). Raw sequence data were also uploaded to the NCBI pathogen detection pipeline for comparison to other publicly available genomes within 50 SNPs (National Center for Biotechnology Information (NCBI), 2025), defined herein as a “cluster”, to enable quick and simple checks for clustering within a public database. After NCBI processing completion, each isolate was periodically checked for SNP clustering with other isolates in the database. If clustering was observed, the clusters were analyzed for the purpose of the submitter’s infection prevention efforts, as well as for clustering with human isolates. In order for an investigation of a human-animal cluster to proceed it would need to meet two criteria: (1) a close genetic relationship and (2) relatively close geographic and temporal relationships. Specifically, while the NCBI pipeline will cluster isolates together if within 50 SNPs, we limited our investigations of human-animal clusters to those with smaller SNP differences (approximately ≤ 15–20 SNPs) regardless of bacterial species. Biosample information was used to confirm that both human and animal isolates were from relatively close geographic regions (defined as within the same state or a bordering state) and temporally close (isolated within 1 year of each other).

Clustering human and animal isolates with similar collection dates and geographical regions were then presented to the PGCoE CPO working group to determine if the potential connections warranted further investigation. For example, if a human and animal cluster was <15 SNPs apart, but the isolates were uploaded retrospectively and collected >2 years prior, it may have been decided not to investigate that cluster further. When the timeline and SNP distance did align, the PGCoE CPO working group reached out to HAI epidemiologists in the associated jurisdiction’s health department to gather more information about the human associated with the clustering isolate through routine public health interventions and to determine if they had capacity and willingness to conduct additional investigations. All discussions, data sharing, and investigations were conducted within regional and federal privacy regulations as dictated by the consulting public health department.

## 3 Successes

Over the past 2 years, the One Health model described herein resulted in identification of eight clusters containing both animal- and human-derived isolates with variable SNP distances (0–11 SNPs). A key cluster investigated through this collaboration identified a genetic link between animal and human isolates on the NCBI pathogen detection pipeline and was previously described (DeStefano et al., 2025b). Briefly, the model of investigation described above linked three genetically related human *E. coli* ST162 isolates harboring *bla*_NDM-5_ to a simultaneous, but separate, CPO investigation at a Massachusetts veterinary hospital. The Massachusetts State Public Health Department was contacted by the PGCoE CPO working group when the NCBI pathogen detection pipeline demonstrated isolate clusters with 0–10 SNPs between humans and animals. Public health interviews with human case patients prior to the identification of the animal isolates in this cluster failed to identify any epidemiological links between the human cases. Once follow-up interviews that included questions related to pet exposure were performed, it was revealed that all three human case patients had pets treated at the same veterinary hospital prior to their own diagnosis.

Our successes are not limited to identification of human-animal clusters. The approach has significantly increased WGS data available to describe companion animal CPE isolates. In the process of examining the relatedness of CPO isolates from animals and humans, we sequenced 78 canine and 16 feline CPO isolates (34 *E. coli*, 29 *Klebsiella* spp., 29 *Enterobacter* spp., 2 *Citrobacter freundii*). Key MLST and carbapenemase genes identified included *E. coli* ST162 and ST410 with *bla*_NDM-5_, *K. pneumoniae* ST11 with *bla*_NDM-5_, *K. pneumoniae* ST307 with *bla*_NDM-5_ or *bla*_NDM-7_, *E. cloacae* ST171 with *bla*_NDM-7_, and *E. cloacae* ST114 with *bla*_NDM-5_. Additional carbapenemase enzyme types such as VIM, KPC, and OXA-48 were also identified, but at much lower rates. Even if sequencing results did not immediately result in an animal-human cluster to explore, they were useful to identify hospital-acquisition events during suspected outbreaks for some veterinary practices. Of note, most isolates, and all of the human-animal clusters, were only associated with *bla*_NDM_ genes, which were recently reported to have increased by over 460% among humans in the USA from 2019 to 2023 (Rankin et al., 2025).

Not only has the PGCoE collaboration enabled expansion of WGS of CPOs in companion animals, but it has also allowed us to close gaps in information available about CPOs and WGS, in general. Identifying clusters comprised of isolates from animals and humans allowed for a communication network to develop between the veterinary clinical laboratories, public health laboratories and departments, and submitting veterinarians. In addition to the data collection tools created, education materials on WGS for health professionals (physicians and veterinarians) and the public are being developed during this process. Education materials often lack guidance on what situations may warrant requesting WGS as well as the logistics, such as laboratory communication, cost, turnaround time, and how the information could benefit the healthcare provider or veterinarian. As WGS becomes more accessible, such resources will be increasingly valuable.

Investigation of these clusters has also increased communication with veterinary practitioners and raised awareness of the problem of CPOs and potential clinical impact on veterinary patients. For example, in the course of one investigation that did not result in elucidation of the relationship between genetically linked animal and human isolates, the veterinary practice partnered with the state health department to apply interventions to mitigate ongoing CPO spread within their facility.

## 4 Challenges

Of all the human and animal clusters identified and follow-up investigations performed, only the Massachusetts cluster described above yielded results that clearly suggested direct transmission between humans and animals. Many factors had to align for that investigation to be successful. Clinicians at the Massachusetts veterinary hospital identified a potential outbreak and worked with the CREATE project to mitigate the spread of CPOs. As part of this response, the animal isolates were sequenced. In addition, the public health department had already started investigating the relationship between human-sourced isolates in the cluster, and the patients involved were willing to participate in the public health interviews.

Most investigations were not as fruitful as detailed in **Table 1**. The main challenges included: limitations to timely WGS, capacity of public health professionals in jurisdictions where clusters are detected, and willingness of affected individuals to participate in follow-up interviews.

**Table 1:** Illustrative examples of human-animal isolate clusters identified using this published framework.

Cluster	Organism	Carbapenemase	MLST	# human isolates	# animal isolates	SNP range	Investigated?/ outcome
A PDS000073337	*E. coli*	NDM-5	361 (Achtman)	1	1 dog	11	Yes; investigation halted due to lack of health department bandwidth
B PDS000107059	*E. coli*	NDM-5	410 (Achtman)	1	1 dog	13	No; geographic/temporal distance
C PDS000127273	*K. pneumoniae*	NDM-5	307	1	7 dogs 1 cat	4-8	Yes; human interviewed but was of low yield
D PDS000149463	*E. coli*	NDM-5	162 (Achtman)	3	10 dogs 1 cat	0-11	Yes; Massachusetts cluster
E PDS000180158	*E. hormaechei*	NDM-5	114	1	1 cat	5	Yes; human declined interview request
F PDS000180176	*K. pneumoniae*	NDM-5	147	1	1 dog	20	No; geographic/temporal distance
G PDS000097502	*K. pneumoniae*	NDM-5	11	1	6 dogs	7-16	No; geographic/temporal distance
H PDS000175170	*K. pneumoniae*	NDM-7	307	1	4 dogs 5 cats	5-24	Yes; investigation halted due to lack of health department bandwidth

The “PDS” number represents the “SNP cluster” which can be accessed via NCBI Pathogen Detection. This table only includes data from isolates available and assessed at the time of investigation but real-time updated clusters on NCBI Pathogen Detection may include additional isolates or data.

Some (3/8) human-animal clusters could not be investigated due to the retrospective timing of WGS. Prior to initiation of this funded project, there was no established sequencing infrastructure for CPOs isolated from companion animals. As such, identifying real-time connections was infeasible and not worth spending epidemiologic resources. Another challenge is the limited bandwidth of public health and veterinary professionals. A cluster of *K. pneumoniae* ST30 harboring *bla*_NDM-7_ was identified from one state in the northeast region of the United States. In this cluster, one human urine isolate was found with 5–24 SNPs from 9 animal isolates (4 dogs and 5 cats). Another cluster of *E. coli* ST361 harboring *bla*_NDM-5_ included a dog isolate that was 11 SNPs from a human urine isolate in the same state. These clusters suggested a potential connection due to geographic and temporal relatedness, so the public health department was contacted. Short-staffing and loss of federal funding prevented the state’s HAI program from conducting additional investigations.

An investigation into *Enterobacter hormaechei* ST114 isolates harboring *bla*_NDM-5_ from another northeast state also had genetic links between human and animal isolates, but failed to elucidate potential connections. In this case, an isolate was obtained from a cat seen at a veterinary practice in the same state as a human urine isolate sequence also collected in 2023. Given their geographic and temporal proximity, the department of health was contacted. The health department contacted the associated individual, but the person declined to be interviewed and the investigation concluded.

Lastly, another investigation involved an outbreak of *K. pneumoniae* ST307 harboring *bla*_NDM-5,_ including a single human isolate and 8 companion animals (7 dogs and 1 cat), seen at the same veterinary practice. Isolates were 4–8 SNPs apart at the time of investigation. Geographic and temporal proximity of these human and animal isolates led to collaboration with the public health department. The human disclosed that their pet had received veterinary care, however, it was not at the clinic of interest in this investigation. This example highlights the limited visibility we have on the overall epidemiology of CPOs in companion animal populations.

One technical limitation to this work includes the use of only short-read sequencing. This approach is helpful when investigating single species outbreaks. Short-read sequencing results in draft genomes with multiple contigs instead of fully closed genomes obtained from long-read sequencing, which would enable reliable identification of plasmids. Therefore, short-read sequencing does not allow for investigations regarding the role of mobile genetic elements such as plasmids within an outbreak (Perreten and Endimiani, 2025; Zhao et al., 2023). There is likely significant value of the use of long-read sequencing or hybrid approaches to investigate potential linkages due to plasmid spread instead of clonal expansion of a single species. This would currently require significant additional bioinformatic support and strategies for our approach, but as pipelines and technologies evolve, then it is likely to become more feasible.

## 5 Conclusions

The One Health approach, which integrates human, animal and environmental dynamics, is critical to advancing our understanding and efforts to control AR. Our model for investigation of CPE spread between humans and companion animals offers a unique approach to One Health investigations. It has helped to explore spread in at least one otherwise hidden cluster of community-associated infections in humans. However, as demonstrated by the number of clusters identified that could not be further explored, there are several structural barriers that we face in untangling the complex epidemiology. As with many public health endeavors, funding and human capital resources are often limited. One Health investigations can be costly in both dollars and in time. Resources need to be made more widely available or more easily shareable to public health or animal health authorities to support this work and formalize networks. For example, carbapenemase testing is often available in public health laboratories but has been shown to be lacking in veterinary health laboratories (Waltenburg et al., 2022), and there is a need to either share knowledge or testing capacity across these spaces. Other examples of capacity needs may include specimen retention, bioinformatic support, and dedicated personnel time.

When capacity building, resources to conduct WGS on veterinary isolates in a timely fashion should also specifically be included. The identification of several closely related human and animal cases in retrospect speaks to potentially matching cases in real-time. Cases can then be more efficiently investigated, connections made, and interventions implemented to prevent additional spread. There are significant costs associated with this sequencing, and jurisdictions should identify which resources are necessary to conduct these investigations prior to having a significant or urgent need.

Additionally, given that this work requires linking One Health data, there is a need to rely on either previously collected data or interviews with CPO positive humans to make connections which often lack information on animal exposure. Veterinary health information is rarely available to human or public health professionals, and reporting systems are rudimentary compared to centralized or automated systems available in human health. In one case described above, the affected person was not willing to participate in the investigation and our ability to further investigate was halted. Like other public health challenges, it is important to build trust with communities and emphasize the importance of yielding attention to AR. Trust is particularly important in the context of the human-animal bond and impacts that investigations may have on that bond.

Our approach demonstrates the need for robust and active collaborations across veterinary, human, and public health sectors to comprehensively assess and track the major challenge that AR bacteria such as CPE present. In addition, review of requirements to report CPOs identified in veterinary laboratories (specifically reference laboratories) to health departments would be of benefit, including potential submission of associated isolates. In our experience, the connection between HAI/AR public health teams, veterinary public health professionals, and veterinary clinicians and diagnosticians is highly variable between jurisdictions, but collaboration is crucial as demonstrated here. Resources for public health testing of animals with CPE also need to be made available more broadly, particularly as outbreaks involving both humans and animals are identified. It is critical to extend public health efforts into veterinary health settings to prevent the spread of CPE within animals and also subsequently to (or back to) humans.

## Data Availability

The original contributions presented in the study are included in the article/**Supplementary Material**. Further inquiries can be directed to the corresponding author.
