# Supplementary material for: Tracking spread of carbapenemase-producing Enterobacterales between humans and companion animals: successes and challenges
Source: Front Cell Infect Microbiol. 2026 Jan 20;15:1730592. doi: 10.3389/fcimb.2025.1730592 (PMC12864382; doi:10.3389/fcimb.2025.1730592)
Supplement: Supplementary file 1 [file Table1.docx]

| **Biosample** | **Host species** | **Organism** | **Carbapenemase** | **MLST** | **Source** |
| --- | --- | --- | --- | --- | --- |
| SAMN38336109 | Cat | *K. pneumoniae* | NDM-5 | 307 | Wound |
| SAMN38336101 | Dog | *K. pneumoniae* | NDM-5 | 76 | Feces |
| SAMN38336102 | Dog | *E. coli* | NDM-5 | 162 (Achtman) | Feces |
| SAMN38336103 | Dog | *E. coli* | NDM-5/OXA-48 | 162 (Achtman) | Feces |
| SAMN38336105 | Dog | *E. coli* | NDM-5 | 162 (Achtman) | Feces |
| SAMN38336106 | Cat | *E. coli* | NDM-5 | 162 (Achtman) | Feces |
| SAMN38336114 | Dog | *E. coli* | NDM-5 | 162 (Achtman) | Feces |
| SAMN38336118 | Dog | *E. coli* | NDM-5 | 162 (Achtman) | Feces |
| SAMN38336104 | Dog | *E. cloacae complex* | NDM-7 | 171 | Feces |
| SAMN38336108 | Dog | *E. cloacae complex* | NDM-7 | 171 | Feces |
| SAMN38336112 | Dog | *E. cloacae complex* | NDM-7 | 171 | Feces |
| SAMN39998600 | Dog | *E. cloacae complex* | NDM-5 | 171 | Surgical site |
| SAMN38336110 | Dog | *K. pneumoniae* | NDM-5 | 11 | Endotracheal tube site |
| SAMN38336111 | Dog | *E. cloacae complex* | NDM-5 | 88 | Endotracheal tube site |
| SAMN38336115 | Dog | *K. pneumoniae* | KPC-3 | 15 | Endotracheal wash |
| SAMN38336116 | Dog | *E. coli* | KPC-3 | N/A | Endotracheal wash |
| SAMN39324480 | Cat | *K. pneumoniae* | NDM-5 | 11 | Urine |
| SAMN38336117 | Dog | *K. pneumoniae* | NDM-5 | 11 | Pleural fluid |
| SAMN38336119 | Dog | *K. pneumoniae* | NDM-5 | 11 | Surgical site |
| SAMN39998599 | Dog | *K. oxytoca* | KPC-2 | 169 | Blood |
| SAMN38336107 | Dog | *E. coli* | NDM-5 | 410 (Achtman) | Feces |
| SAMN33902370 | Dog | *E. coli* | NDM-7 | 2137 (Achtman) | Feces |
| SAMN38336113 | Cat | *E. cloacae complex* | NDM-7 | 171 | Endotracheal tube site |
| SAMN38501005 | Dog | *K. pneumoniae* | NDM-5 | 307 | Urine |
| SAMN39998604 | Dog | *E. cloacae complex* | NDM-7 | 171 | Feces |
| SAMN39998602 | Dog | *E. cloacae complex* | NDM-7 | 171 | Feces |
| SAMN39998603 | Dog | *E. cloacae complex* | NDM-7 | 171 | Feces |
| SAMN39998605 | Dog | *E. cloacae complex* | NDM-7 | 171 | Feces |
| SAMN39998606 | Dog | *E. cloacae complex* | NDM-7 | 171 | Feces |
| SAMN39324489 | Dog | *E. cloacae complex* | NDM-7 | 171 | Feces |
| SAMN39324493 | Dog | *E. cloacae complex* | NDM-7 | 171 | Feces |
| SAMN39324491 | Dog | *E. coli* | NDM-5 | 361 (Achtman) | Feces |
| SAMN39324488 | Cat | *E. coli* | KPC-3 | 1859 (Achtman) | Feces |
| SAMN39324492 | Dog | *K. pneumoniae* | NDM-7 | 307 | Urine |
| SAMN39324490 | Dog | *E. coli* | NDM-5 | 410 (Achtman) | Feces |
| SAMN39324484 | Dog | *K. pneumoniae* | NDM-5 | 11 | Feces |
| SAMN39324481 | Dog | *E. coli* | NDM-5 | 372 (Achtman) | Feces |
| SAMN39324482 | Dog | *K. pneumoniae* | NDM-5 | 11 | Feces |
| SAMN39324483 | Dog | *E. coli* | NDM-5 | 162 (Achtman) | Feces |
| SAMN39998601 | Dog | *E. cloacae complex* | NDM-5 | 171 | Feces |
| SAMN39324487 | Dog | *E. coli* | VIM-4 | 224 (Achtman) | Feces |
| SAMN39324485 | Dog | *E. coli* | VIM-4 | 224 (Achtman) | Feces |
| SAMN39324486 | Dog | *C. freundii* | NDM-5 | 22 | Feces |
| SAMN40717021 | Cat | *E. cloacae complex* | NDM-5 | 114 | Feces |
| SAMN40717019 | Dog | *E. cloacae complex* | OXA-48 | 78 | Feces |
| SAMN40419212 | Dog | *E. coli* | NDM-5 | 162 (Achtman) | Feces |
| SAMN40717015 | Dog | *K. pneumoniae* | NDM-5 | 147 | Urine |
| SAMN40717017 | Dog | *E. coli* | KPC-3 | 2585 (Achtman) | Feces |
| SAMN40717020 | Dog | *E. coli* | NDM-5 | 162 (Achtman) | Feces |
| SAMN40717016 | Cat | *K. pneumoniae* | NDM-5 | 11 | Urine |
| SAMN40190961 | Dog | *E. coli* | NDM-5 | 410 (Achtman) | Wound |
| SAMN40190957 | Dog | *K. pneumoniae* | NDM-7 | 307 | Feces |
| SAMN40190959 | Dog | *E. cloacae complex* | NDM-7 | 171 | Feces |
| SAMN40190960 | Cat | *E. cloacae complex* | NDM-7 | 171 | Feces |
| SAMN40717018 | Dog | *E. coli* | NDM-5 | 457 (Achtman) | Feces |
| SAMN41423297 | Cat | *E. coli* | NDM-5 | N/A | Bladder mucosa |
| SAMN40190958 | Dog | *E. coli* | NDM-5 | 224 (Achtman) | Feces |
| SAMN41130365 | Dog | *E. cloacae complex* | NDM-7 | 171 | Feces |
| SAMN41130366 | Dog | *E. cloacae complex* | NDM-7 | 171 | Feces |
| SAMN41130392 | Dog | *E. cloacae complex* | NDM-7 | 171 | Feces |
| SAMN41130367 | Dog | *K. pneumoniae* | NDM-5 | 11 | Feces |
| SAMN41130368 | Cat | *E. coli* | NDM-5 | 2003 (Achtman) | Feces |
| SAMN41130369 | Dog | *E. cloacae complex* | NDM-7 | 171 | Feces |
| SAMN42123160 | Dog | *K. pneumoniae* | NDM-5 | 307 | Urine |
| SAMN42123161 | Dog | *E. coli* | NDM-5 | 68 (Achtman) | Feces |
| SAMN42123162 | Dog | *E. cloacae complex* | NDM-7 | 171 | Urine |
| SAMN42123163 | Dog | *E. coli* | NDM-5 | 162 (Achtman) | Feces |
| SAMN42123164 | Dog | *C. freundii* | NDM-5 | 580 | Urine |
| SAMN42533176 | Dog | *E. cloacae complex* | OXA-48 | 109 | Feces |
| SAMN42533473 | Dog | *E. coli* | OXA-484 (OXA-48 variant) | 101 (Achtman) | Feces |
| SAMN42533177 | Dog | *E. cloacae complex* | NDM-5 | 114 | Feces |
| SAMN42533178 | Dog | *E. coli* | NDM-7 | 162 (Achtman) | Feces |
| SAMN42533471 | Dog | *E. coli* | NDM-5 | 491 (Achtman) | Feces |
| SAMN42533472 | Dog | *E. coli* | NDM-5 | 162 (Achtman) | Feces |
| SAMN43368438 | Cat | *K. pneumoniae* | NDM-7 | 307 | Abominal fluid |
| SAMN43368446 | Dog | *E. coli* | NDM-7 | 224 (Achtman) | Etube site |
| SAMN43368439 | Cat | *K. pneumoniae* | NDM-7 | 307 | Feces |
| SAMN43368440 | Dog | *E. cloacae complex* | NDM-5 | 114 | Feces |
| SAMN43368441 | Dog | *E. coli* | NDM-5 | 1262 (Achtman) | Feces |
| SAMN43368445 | Dog | *K. pneumoniae* | NDM-5 | 11 | Surgical site |
| SAMN43368443 | Cat | *K. pneumoniae* | NDM-7 | 307 | Abdominal fluid |
| SAMN43368444 | Dog | *E. cloacae complex* | NDM-7 | 171 | Feces |
| SAMN44716452 | Dog | *K. pneumoniae* | NDM-5 | 307 | Wound |
| SAMN44716453 | Dog | *E. coli* | OXA-48 (OXA-1205) | 746 (Achtman) | Feces |
| SAMN44740054 | Dog | *K. pneumoniae* | NDM-5 | 11 | Surgical site |
| SAMN44716454 | Cat | *K. pneumoniae* | NDM-7 | 307 | Feces |
| SAMN44716455 | Dog | *E. coli* | NDM-5 | 410 (Achtman) | Feces |
| SAMN44716456 | Dog | *K. pneumoniae* | NDM-7 | 17 | Feces |
| SAMN44716457 | Dog | *E. cloacae complex* | NDM-7 | 171 | Feces |
| SAMN44716458 | Cat | *E. cloacae complex* | NDM-7 | 171 | Urine |
| SAMN44716459 | Cat | *K. pneumoniae* | NDM-5 | 307 | Feces |
| SAMN44716460 | Dog | *K. pneumoniae* | NDM-5 | 307 | Tracheal aspirate |
| SAMN44716461 | Dog | *K. pneumoniae* | NDM-5 | 307 | Urine |
| SAMN44716462 | Dog | *K. pneumoniae* | NDM-5 | 307 | Urine |
