# Supplementary material for: Tracking spread of carbapenemase-producing Enterobacterales between humans and companion animals: successes and challenges
Source: Front Cell Infect Microbiol. 2026 Jan 20;15:1730592. doi: 10.3389/fcimb.2025.1730592 (PMC12864382; doi:10.3389/fcimb.2025.1730592)
Supplement: Supplementary file 2 [file DataSheet1.pdf]

REDCap

Companion Animal CPO Pathogen Genomics

Epi & Clinical Information

Record ID

Patient ID/MRN: _____
Isolate ID: _____

State of residence

County of residence

Clinical Information

Is a primary problem list available? ○ Yes ○ No ○ Unknown

Specify primary problems

Did the animal receive immunosuppressive medication(s)? ○ Yes ○ No ○ Unknown

Specify immunosuppressive medication(s)

Reason for presentation for care when specimen was collected

- ○ Inpatient (surgical)
- ○ Inpatient (medical)
- ○ Inpatient (boarding)
- ○ Outpatient (surgical)
- ○ Outpatient (medical/wellness visit)
- ○ Colonization screening

How many times was the animal hospitalized in the year prior to specimen collection?

Did animal receive antimicrobial drugs in the 6 months before specimen collection? ○ Yes ○ No ○ Unknown

List the antimicrobial(s) received in the 6 months before specimen collection.

Note: If the animal received the same drug more than one once in a time frame, just list it once. If the animal received the drug in multiple time frames, record in each (e.g., If a dog received cefpodoxime for 7 days 20 days ago and for 10 days 45 days ago, cefpodoxime would be listed in both the 30-day and 90-day time frames).

Within 30 days of specimen	31-90 days before specimen	91 days-6 months before specimen

Patient outcome

- ○ Alive
- ○ Euthanized
- ○ Died
- ○ Unknown

Date of death M-D-Y

Epi Information

Does the history indicate exposure to:

**Jerky treats**	
**Pig's ears**	
**Bully sticks**	
**Raw food**	Yes

Raw foods:

**Poultry**	
**Pork**	
**Beef**	
**Rabbit**	
**Fish**	
**Other**	
**Prepared at**	

Does the history indicate exposure to:

**Dog park**	
**Veterinary clinic**	
**Animal day care**	
**Animal boarding**	
**Animal grooming**	
**Human hospital**	
**Human long-term care**	

Has the animal traveled in the past year?
- Yes
- No
- Unknown

Did the animal travel within 30 days of specimen collection?
- Yes
- No
- Unknown

Travel locations and dates

Was the animal imported from another country?
- Yes
- No
- Unknown

Animal's country of origin

Is this a working animal?
- Yes
- No
- Unknown

If able, please list the type of tasks the animal is trained to provide.

Form Status

Complete? Incomplete
