# Supplementary material for: Tracking spread of carbapenemase-producing Enterobacterales between humans and companion animals: successes and challenges
Source: Front Cell Infect Microbiol. 2026 Jan 20;15:1730592. doi: 10.3389/fcimb.2025.1730592 (PMC12864382; doi:10.3389/fcimb.2025.1730592)
Supplement: Supplementary file 3 [file DataSheet2.pdf]

Isolate Information

Record ID

Collecting facility type
- ○ Tertiary care facility (e.g. veterinary teaching hospital)
- ○ General practice
- ○ Referral practice
- ○ Animal rescue/shelter
- ○ Other
- ○ Unknown

Submitting laboratory ____________

Patient Signalment

Species
- ○ Canine
- ○ Feline
- ○ Other
- ○ Unknown

Breed ____________

Age (at time of specimen collection) ____________

Age units ○ Weeks ○ Months ○ Years

Unknown age ☐

Sex
- ○ Male (intact)
- ○ Male (neutered)
- ○ Female (intact)
- ○ Female (spayed)
- ○ Unknown

Patient ID/MRN ____________

Isolate Information

Isolate/specimen ID ____________

Collection date ____________ M-D-Y

Date of isolate submission to MDH ____________ M-D-Y

No isolate available ☐

Clinical or screening isolate
- ○ Clinical
- ○ Screening
- ○ Unknown

Collection site
- ○ Blood
- ○ Bone
- ○ Bronchoalveolar lavage
- ○ CSF
- ○ Internal body site (specify)
- ○ Joint/synovial fluid
- ○ Muscle
- ○ Pericardial fluid
- ○ Peritoneal fluid
- ○ Pleural fluid
- ○ Sputum
- ○ Swab (specify)
- ○ Tracheal aspirate
- ○ Urine
- ○ Other site
- ○ Unknown

Type of organism
- ○ CRE
- ○ CRAB
- ○ CRPA
- ○ Other

Organism ______

MDH lab accession number ______

Date received at MDH ______ M-D-Y

Microbiological Information

Carbapenemase tests conducted
- ☐ None
- ☐ mCIM
- ☐ Carba NP
- ☐ Carba 5
- ☐ Other phenotypic method
- ☐ PCR
- ☐ Whole-genome sequencing

Other phenotypic method

______

mCIM test result

○ Positive ○ Negative ○ Indeterminate ○ Unknown

Carba NP test result

○ Positive ○ Negative ○ Indeterminate ○ Unknown

Carba 5 test results

**NDM**	○ Positive ○ Negative ○ Indeterminate
**KPC**	○ Positive ○ Negative ○ Indeterminate
**VIM**	○ Positive ○ Negative ○ Indeterminate
**IMP**	○ Positive ○ Negative ○ Indeterminate
**OXA-48**	○ Positive ○ Negative ○ Indeterminate

_____ test result

○ Positive ○ Negative ○ Indeterminate ○ Unknown

Molecular test results

**NDM**	○ Positive ○ Negative ○ Indeterminate ○ Unknown ○ Not tested
**KPC**	○ Positive ○ Negative ○ Indeterminate ○ Unknown ○ Not tested
**VIM**	○ Positive ○ Negative ○ Indeterminate ○ Unknown ○ Not tested
**IMP**	○ Positive ○ Negative ○ Indeterminate ○ Unknown ○ Not tested
**OXA**	○ Positive ○ Negative ○ Indeterminate ○ Unknown ○ Not tested
	Specify OXA gene(s)
**Other gene**	○ Positive ○ Negative ○ Indeterminate ○ Unknown ○ Not tested
	Specify other carbapenemase gene

PCR test type

○ Commercial test ○ Laboratory-developed test (LDT)

Carbapenem resistance

☐ Imipenem
☐ Meropenem
☐ Ertapenem
☐ Other
☐ Unknown

Specify other carbapenem

If available, please upload unsuppressed AST results here.

Are you willing and able to provide clinical or epidemiological information at this time?

○ Yes (enables additional survey upon submission)
○ No

Form Status

Complete? Incomplete
